# Multi-Cycle Dosimetry of the Albumin-Binding Prostate-Specific Membrane Antigen Ligand [^177^Lu]Ludotadipep in Metastatic Androgen Pathway Modulator-Resistant Prostate Cancer with Adjusted Red Marrow Modeling

**DOI:** 10.3390/pharmaceutics18101328

**Published:** 2026-10-20

**Authors:** Seunggyun Ha, Joo Hyun O, Chansoo Park, Sun Ha Boo, Dongho Shin, Hyong Woo Moon, Ie Ryung Yoo, In-Ho Kim, Ji Youl Lee

**Affiliations:** 1 Department of Radiology, Division of Nuclear Medicine, College of Medicine, The Catholic University of Korea, Seoul 06591, Republic of Korea; seunggyun.ha@gmail.com (S.H.);; 2 Research Institute of Labeling, FutureChem Co. Ltd., Seoul 06591, Republic of Korea; 3 Department of Urology, College of Medicine, The Catholic University of Korea, Seoul 06591, Republic of Korea; 4 Department of Internal Medicine, Division of Medical Oncology, College of Medicine, The Catholic University of Korea, Seoul 06591, Republic of Korea

**Keywords:** [^177^Lu]Ludotadipep, PSMA, multicycle dosimetry, red marrow, albumin

## Abstract

**Purpose:** This dosimetry study aimed to evaluate the organ-absorbed doses and safety profile of multiple doses of [^177^Lu]Ludotadipep, a novel PSMA-targeted radiopharmaceutical with an albumin-binding motif, in patients with metastatic androgen pathway modulator-resistant (mAPMR) prostate cancer. **Methods:** Twenty patients received a total of 68 cycles of [^177^Lu]Ludotadipep (3.70 GBq/cycle, up to six cycles, 8-week intervals), and dosimetry analysis was done for 67 cycles. Organ-absorbed doses were estimated using planar and SPECT/CT imaging and blood-based methods. Red marrow dose was calculated using a red marrow-to-blood activity concentration ratio (RMBLR) derived from a hematocrit-adjusted model for large molecules and cross-checked by comparison with data derived from quantitative SPECT/CT images and blood samples. **Results:** The hematocrit-adjusted RMBLR of 0.28 was consistent with physiologic expectations for albumin-bound agents for marrow dosimetry. The highest absorbed dose coefficients in the first cycle were in the salivary glands and kidneys (1.22 ± 0.530 and 0.674 ± 0.330 Gy/GBq, respectively), while the red marrow dose (0.0533 ± 0.0115 Gy/GBq) was lower. Summing the cycle-specific mean absorbed dose coefficients and multiplying by 3.70 GBq per cycle yielded projected six-cycle absorbed doses of 18.1 Gy for kidneys, 15.3 Gy for salivary glands, and 1.28 Gy for red marrow, all of which were below the reference dose constraints. Salivary gland dose coefficients declined significantly over repeated cycles (*p*  <  0.001), possibly from radiation-induced tissue damage. **Conclusion:** [^177^Lu]Ludotadipep demonstrates a favorable dosimetric safety profile across multiple cycles, up to six cycles. Application of an appropriately adjusted RMBLR for albumin-binding behavior allowed for reproducible red marrow dose estimation.

## 1. Introduction

Prostate-specific membrane antigen (PSMA)-targeted radionuclide therapy (TRT) is now a standard treatment option for patients with metastatic androgen pathway modulator-resistant (mAPMR) prostate cancer, following the success of [^177^Lu]Lu-PSMA-617 in the VISION trial [1]. Efforts to enhance outcomes continue with strategies to increase tumor uptake and optimize the pharmacokinetics of PSMA-targeted agents [2,3,4]. Reversible albumin binding can prolong blood residence and increase the opportunity for tumor uptake; however, prolonged circulation may also increase exposure of the kidneys and red marrow. Clinically investigated albumin-binding strategies include para-substituted phenylbutyrate derivatives, Evans blue derivatives, and ibuprofen-based moieties [5,6,7]. Recent reviews summarize these chemical approaches and their translation to PSMA-targeted radioligand therapy [8,9].

[^177^Lu]Ludotadipep is a novel PSMA-targeting radiopharmaceutical engineered with an iodophenylbutyrate-derived albumin-binding motif to extend the systemic circulation time and thus increase tumor accumulation [10]. The phase 1 dosimetry study showed that [^177^Lu]Ludotadipep can achieve high tumor absorbed doses while maintaining an acceptable safety profile, identifying the kidneys and red marrow as the primary dose-limiting organs [11]. Further evaluation in multi-cycle treatment settings was necessary to check the reproducibility of the initial data and to assess cumulative organ exposure to ensure the safety of repeated administrations. Accurate dosimetry evaluation of the red marrow was considered a special challenge, particularly as this agent has a biodistribution altered by albumin binding [5,11]. In estimating the red marrow-to-blood activity concentration ratio (RMBLR) for albumin-binding molecules, it is important to consider their limited distribution within the red marrow extracellular fluid (ECF) compartment, which is composed of intravascular plasma and interstitial fluid. The red marrow ECF fraction (RMECFF) of albumin was experimentally measured to be 19% of the marrow volume in rabbits [12,13]. Albumin passes the marrow sinusoidal walls quite freely, and the albumin-accessible marrow space was approximately 84% of the inulin-accessible extracellular space [12]. The estimated RMBLR for albumin-binding molecules would be comparable to the suggested RMBLR of monoclonal antibodies, and much lower than that of 1, suggested for small peptides [13,14,15]. Applying the commonly used RMBLR of 1 for large molecules could potentially overestimate the radiation risk to the marrow and lead to underestimation of the safe therapeutic dose.

The primary focus of this report was the dosimetric assessment of red marrow safety during repeated [^177^Lu]Ludotadipep administration. In addition to evaluating organ dose coefficients over successive cycles, we examined whether a hematocrit (HCT)-adjusted RMBLR was supported by two complementary approaches using quantitative SPECT/CT and blood measurements. This combination of multi-cycle dosimetry and in vivo evaluation of the marrow-to-blood relationship is an extension from the previous single-administration study, in which we used an RMBLR of one.

## 2. Materials and Methods

### 2.1. Participants

This phase II clinical trial was approved by the hospital’s Institutional Review Board (KC22MDSS0440) and the Korean Ministry of Food and Drug Safety. Written informed consent was obtained from all participants and the study was conducted in compliance with the Declaration of Helsinki and local regulations (ClinicalTrials.gov identifier NCT05579184). This study enrolled patients with mAPMR (blood testosterone level ≤ 50 ng/dL) who progressed after standard treatment comprising second-generation androgen pathway-targeted agents (such as enzalutamide or abiraterone), and taxane-based chemotherapy (such as docetaxel and cabazitaxel). Patients considered unsuitable for these treatments by the treating physicians were also eligible. Subjects were screened with [^18^F]Florastamin PSMA PET/CT for unequivocally PSMA-positive lesions [16]. A total of 20 subjects were included and assessed for dosimetry. [^177^Lu]Ludotadipep of 3.70 GBq ± 10% was administered for up to 6 cycles with intervals of 8 ± 2 weeks in each subject and safety assessments were made at 1-, 4-, and 6-weeks post-injection (p.i.).

### 2.2. Radiopharmaceuticals

[^18^F]Florastamin and [^177^Lu]Ludotadipep were manufactured on the day of injection under good manufacturing practice at FutureChem (Seoul, Republic of Korea), as previously described [3,17]. [^18^F]Florastamin was produced with an automated synthesizer (sCUBE, FutureChem, Seoul, Republic of Korea) according to the established radiosynthesis protocol [17]. For [^177^Lu]Ludotad was prepared using an automated synthesis module (miniAllinOne, Trasis, Ans, Belgium; or sCUBE-M, FutureChem, Seoul, Republic of Korea); brieflyipep, no-carrier-added [^177^Lu]L (Isotopia, Indianapolis, IN, USA) uCl3 was combined with the ludotadipep precursor in 1 M sodium acetate/hydrochloric acid buffer (pH 4.88) containing L-ascorbic acid. The mixture was heated at 90 °C for 5–10 min, followed by solid-phase purification with a C18 light cartr (Waters, Milford, MA, USA) idge [11]. Quality control for both radiopharmaceuticals included appearance and particulate assessment, pH, radionuclidic and radiochemical identity, radiochemical and chemical purity, residual solvents, bacterial endotoxins, and sterility testing, as described in the phase 1 report [11].

### 2.3. PET/CT

[^18^F]Florastamin, which has the same primary binding domain consisting of Glu-urea-Lys as that of [^177^Lu]Ludotadipep, was used as the diagnostic radioligand [18,19]. [^18^F]Florastamin PSMA PET/CT was performed at screening and 8 weeks p.i. using three dedicated PET/CT scanners (one of Discovery 710, GE Healthcare, Chicago, IL, USA; two of Biograph Vision 600, Healthineers, Knoxville, TN, USA). Images were acquired 90 min after the intravenous injection of 185 ± 19 MBq of [^18^F]Florastamin. The static images were reconstructed using ordered-subset expectation maximization and point-spread function (PSF) and time-of-flight (TOF) methods (VPFX-S for Discovery 710 and PSF + TOF 4i5s for Vision 600).

### 2.4. Data Acquisition

Before injection of [^177^Lu]Ludotadipep, blank and whole-body transmission scans with ^57^Co flood source were acquired. Post-treatment whole-body emission scans were obtained at 2, 24, 48, 72, and 168 h p.i. Whole-body emission scans were acquired with a medium energy collimator, dual energy window (photopeak window, 187.2–228.8 keV; lower window, 153.9–187.2 keV), and scan speed of 17 cm/min [20]. A point source with known radioactivity containing approximately 3.7 MBq ^177^Lu was placed between the knees at each time point for image calibration. Quantitative SPECT/CT was acquired and used for RMBLR validation. For red marrow dosimetry, 2 mL of peripheral venous sample was obtained from the arm opposite the radiopharmaceutical injection site at 6 time points—3, 15, and 90 min, and 24, 48, and 72 h p.i—adapted from the EANM dosimetry recommendation and based on the biodistribution data from the phase 1 study [15].

After centrifugation, 0.3 mL plasma aliquots were measured in a gamma well counter. Quantitative calibration was performed in accordance with the EANM Dosimetry Committee guidelines [21], using a ^177^Lu reference standard of known activity with the same volume (0.3 mL) as the plasma aliquots. The standard was measured in the same counter, and its known activity (about 3.7 MBq) and measured count rate were used to derive a calibration factor for converting plasma sample count rates into activity. Reference-source decay and aliquot-to-total-volume scaling were accounted for in the activity conversion. Plasma sample count rates were within the range of 1.0 × 10^4^ to 2.0 × 10^6^ counts per minute (cpm). Assuming negligible erythrocyte-associated activity, whole-blood activity concentrations were calculated by multiplying plasma activity concentrations by (1 − HCT).

The WIZARD 2470 (PerkinElmer, Waltham, MA, USA) was used initially and was retired, after which the COBRA 5003 (Packard Instrument, Meriden, CT, USA) was used. Based on the actual counting dates, 27 of 67 evaluable treatment-cycle blood-sampling series (40.3%) were measured with WIZARD and 40 (59.7%) with COBRA. For WIZARD, routine quality control comprised daily background measurements (≤100 cpm) and weekly checks of peak deviation (within ± 5% using ^129^I), energy resolution (≤34% using ^129^I), and counting efficiency (≥74% using ^125^I). For COBRA, the quality-control acceptance criteria were background ≤ 400 cpm, energy resolution ≤ 14% using ^137^Cs, and counting efficiency > 74% using ^125^I.

### 2.5. Dosimetry Analysis

The kidneys, liver, lungs, salivary glands, spleen, and red marrow were the selected source organs. QDOSE software v.1.2.8 (ABX-CRO, Dresden, Germany) was used to draw the region of interest (ROI), to estimate the radioactivity of the ROI, and for fitting the time–activity curves (TAC). The ROI of the source regions other than the red marrow was drawn by sequential automatic segmentation, after co-registration of images for each time point. The two-dimensional activity calculations, including attenuation, scatter, and background corrections for the measurement of activity for each source region ROI, were done by referencing the MIRD pamphlets 16 and 26 [20,22].

### 2.6. RMBLR Estimation and Validation

Red marrow activity was estimated from the blood samples. Assuming that none of the [^177^Lu]Ludotadipep specifically binds to the red marrow cells, the HCT-adjusted RMBLR can be calculated as follows:

$$RMBLR=\;\frac{C_{RM}}{C_{blood}}=\;\frac{0.19}{1-HCT}$$

Here, C denotes activity concentration, RMECFF is the assumed albumin-accessible red marrow extracellular fluid fraction of 0.19. The factor (1-HCT) converts plasma concentration to whole-blood concentration under the assumption of negligible erythrocyte uptake. The cohort-derived value was rounded to 0.28 and applied as a fixed RMBLR for the dosimetry calculation.

Validation used 17 evaluable imaging time points from five patients without perceptible metastases in the lumbar vertebrae or posterior iliac regions. We applied two complementary approaches for checking the computed RMBLR in selected patients. In the combined blood-sampling and SPECT/CT approach, activity measured in the L2-L4 marrow region was extrapolated to total red marrow activity using the reference L2-L4 marrow fraction of 0.067 [21,23,24]. This image-derived total activity was divided by the total marrow activity predicted from the venous whole-blood activity concentration using RMBLR = 1 and the marrow volume used in the dosimetry model. Image and blood activities were compared on a consistent, decay-uncorrected basis:

$$A_{RM,\;SPECT}=\;\frac{A_{L2-L4}}{0.067}$$

$${RMBLR}_{blood+SPECT}=\frac{A_{RM,SPECT}}{C_{blood}\times V_{RM}}$$

Here, V_RM_ denotes the marrow volume used to predict total marrow activity.

In the quantitative SPECT/CT-only approach, the mean standardized uptake value (SUV_mean_) of the trabecular regions of the posterior iliac bones, which are the usual sites for marrow biopsy in clinical practice, was divided by the SUV_mean_ in the superior vena cava (SVC) blood pool on the same image. Because both measurements share the same SUV normalization, this quotient represents the image-derived marrow-to-blood activity concentration ratio:

$${RMBLR}_{QuantSPECT}=\;\frac{{SUVmean}_{posterior\;ilium}}{{SUVmean}_{SVC}}$$

### 2.7. Time–Activity Curve Fitting and Calculation of Absorbed Dose Coefficients

Biexponential TAC fitting was applied to the kidneys, liver, lungs, spleen, and total body, which have peaks at the first time point (2 h p.i.). For salivary glands, which have delayed peaks at second or third time points (24 h or 48 h p.i.), combination of trapezoidal method till the peak and mono- or bi-exponential fitting after the peak was applied. Given the long circulating time of [^177^Lu]Ludotadipep with the albumin binding motif, we applied tri-exponential fitting to the red marrow TAC to reflect all three phases—the rapid clearance, transition, and long tail phases of blood pool activity [11]. In cases where the planned TAC fitting failed, a conservative approach was taken with a combination of the trapezoidal method till the last time point, and mono-exponential fitting with the last 2 or 3 time points, or bi-exponential fitting with the last 4 or 5 time points standing in place of the late phase. Time-integrated activity of the source regions was input into the IDAC 2.1 software v1.01 to calculate the absorbed dose coefficients applying phantom organ masses, except for the red marrow mass which was separately calculated [25].

### 2.8. Statistics

Linear regression analysis was applied for comparison of red marrow and blood pool activity. Mann–Whitney’s U test was applied to test the difference in absorbed dose coefficients between cycle 1, cycle 2/3, and cycle 4/5/6. Statistical significance was defined as *p*-value < 0.05 with Bonferroni’s multiple comparison correction. We assumed an uncorrected *p*-value less than 0.10 to have borderline significance.

## 3. Results

### 3.1. Demographics

Twenty subjects were included, with a mean age of 75.3 ± 9.7 years and mean baseline prostate-specific antigen level of 234.1 ± 244.9 ng/mL. The 20 subjects received 68 administrations of [^177^Lu]Ludotadipep, corresponding to a mean of 3.4 ± 1.3 administrations per subject and a mean cumulative administered activity of 13.0 ± 5.1 GBq.

The third cycle of Subject A01008 was not evaluable because of incomplete data acquisition; therefore, 67 cycles were included in the dosimetric analysis. The numbers of subjects with evaluable dosimetry in cycles 1 through 6 were 20, 19, 14, 8, 5, and 1, respectively.

### 3.2. Estimation and Validation of RMBLR

**Figure 1:**
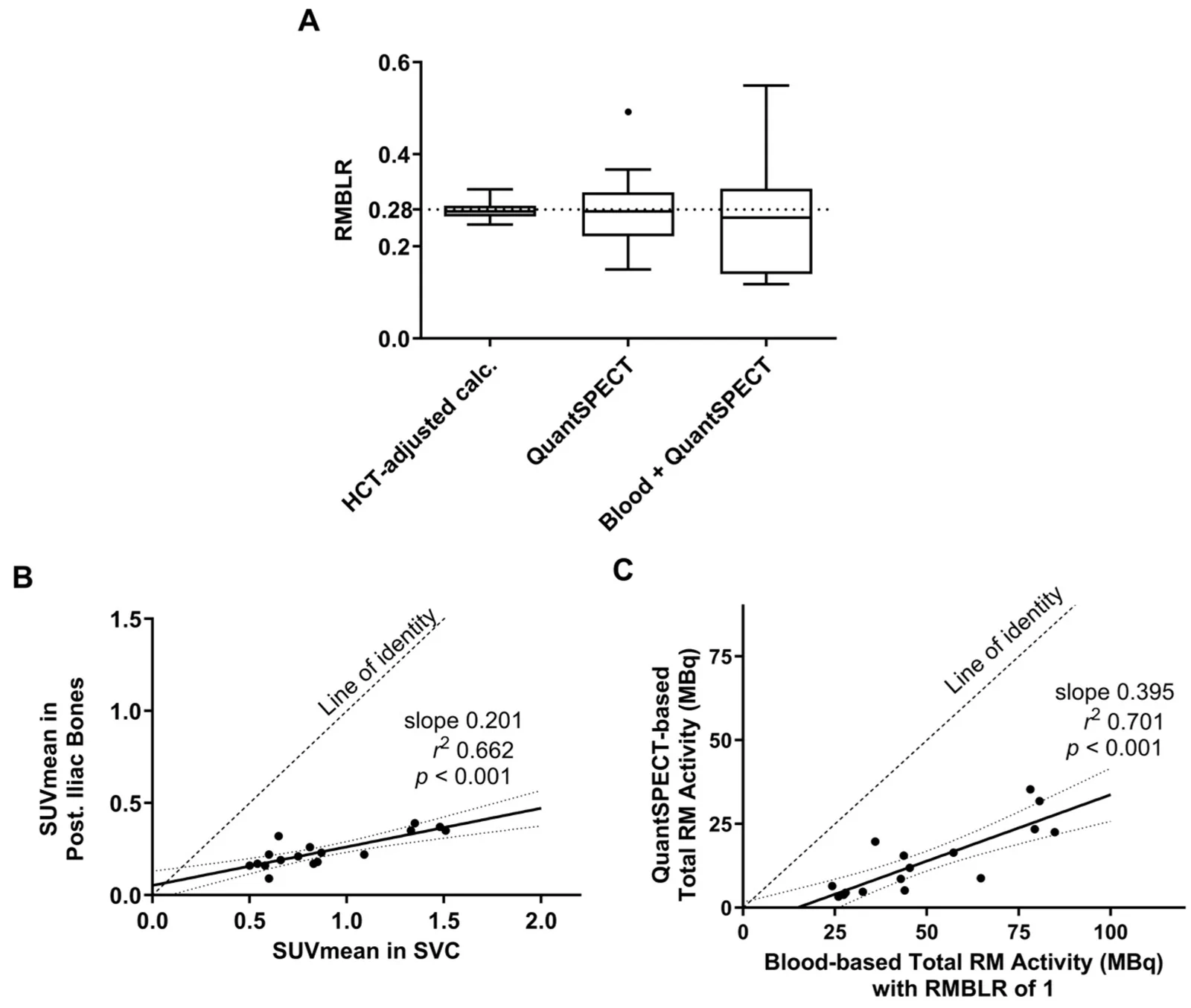
Comparison of red marrow-to-blood activity concentration ratio (RMBLR) values estimated by hematocrit (HCT)-adjusted calculation, quantitative SPECT data (QuantSPECT), and combined blood sample and SPECT data, with the dashed line representing the applied study-specific RMBLR value of 0.28 (**A**). Distribution of measured data was plotted with linear regression analysis for QuantSPECT method (**B**) and combined blood plus QuantSPECT methods (**C**). In panels B and C, the solid lines represent the fitted linear regression lines, the dotted lines indicate the 95% confidence bands, and the dashed lines represent the lines of identity (y = x).

The average HCT at baseline was 0.315 ± 0.040, and the calculated RMBLR value adjusted for the HCT was 0.278 ± 0.017 from all 20 subjects. For the five subjects selected for validation (17 time point data), the RMBLR adjusted for HCT was 0.284 ± 0.010. Using the first method, the SUV_mean_ was measured from the SVC and posterior iliac bones at each time point from the quantitative SPECT images, and the calculated RMBLR was 0.278 ± 0.077. The second method used the venous blood activity and the SPECT images (both decay-uncorrected). The total red marrow activity calculation based on the venous activity while assuming an RMBLR of 1.0 was 48.3 ± 21.6 MBq. The red marrow activity estimated from the L2-4 vertebrae based on the SPECT data was 13.3 ± 10.2 MBq. The study-specific RMBLR was calculated using a combination of the two: L2-4 vertebra red marrow activity estimate/total red marrow activity from venous sample. The average of the study-specific RMBLR values calculated for each time point was 0.252 ± 0.123. Based on these results, we applied a study-specific RMBLR of 0.28 for dosimetry analysis (Figure 1A). The linear regression analysis for the quantitative SPECT data method (Figure 1B) and the blood plus SPECT data method (Figure 1C) showed statistical significance (*r*^2^ 0.66, *p* < 0.001; *r*^2^ 0.70, *p* < 0.001).

### 3.3. Absorbed Dose Coefficient (Gy/GBq) per Cycle

**Figure 2:**
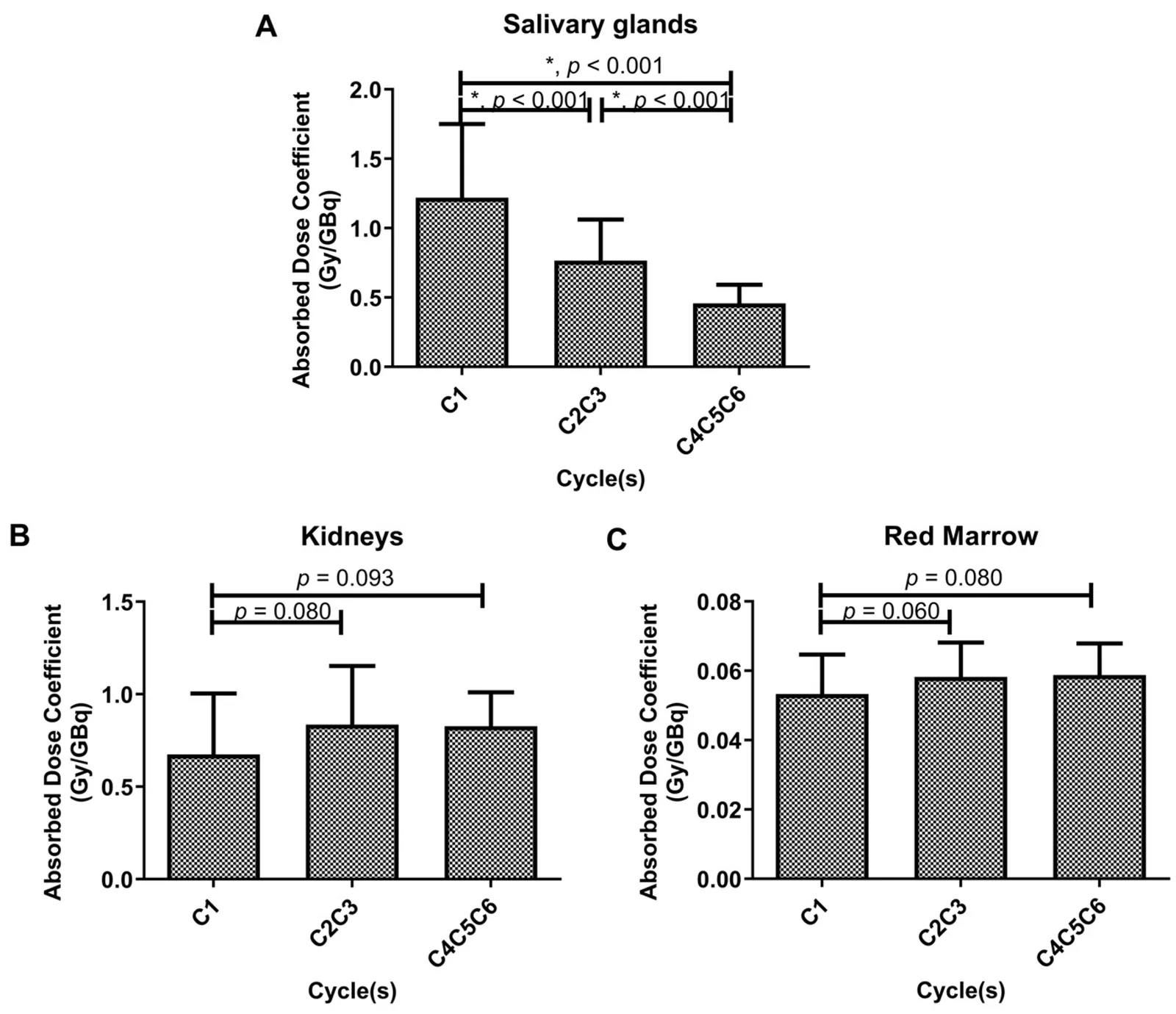
Absorbed dose coefficients over six treatment cycles for the (**A**) salivary glands, (**B**) kidneys, and (**C**) red marrow, showing a significant decrease in the salivary glands and borderline increasing trend in the kidneys and red marrow. C1 denotes cycle 1 (20 administrations), C2C3 denotes cycles 2–3 (33), and C4C5C6 denotes cycles 4–6 (14).

Table 1 presents absorbed dose coefficients of the selected source organs. For the first cycle of [^177^Lu]Ludotadipep administration, the highest absorbed dose coefficient was 1.22 ± 0.530 Gy/GBq in the salivary glands, followed by 0.674 ± 0.330 Gy/GBq in the kidneys. The red marrow demonstrated an absorbed dose coefficient of 0.0533 ± 0.0115 Gy/GBq in the first cycle. The absorbed dose coefficients of the salivary glands decreased significantly over the cycles: 0.765 ± 0.297 Gy/GBq for cycles 2/3 and 0.458 ± 0.135 Gy/GBq for cycles 4/5/6 (Figure 2). Meanwhile, the absorbed dose coefficients of the kidneys, liver, and red marrow showed slightly increasing trends without statistical significance.

### 3.4. Cumulative Absorbed Dose Coefficient

**Figure 3:**
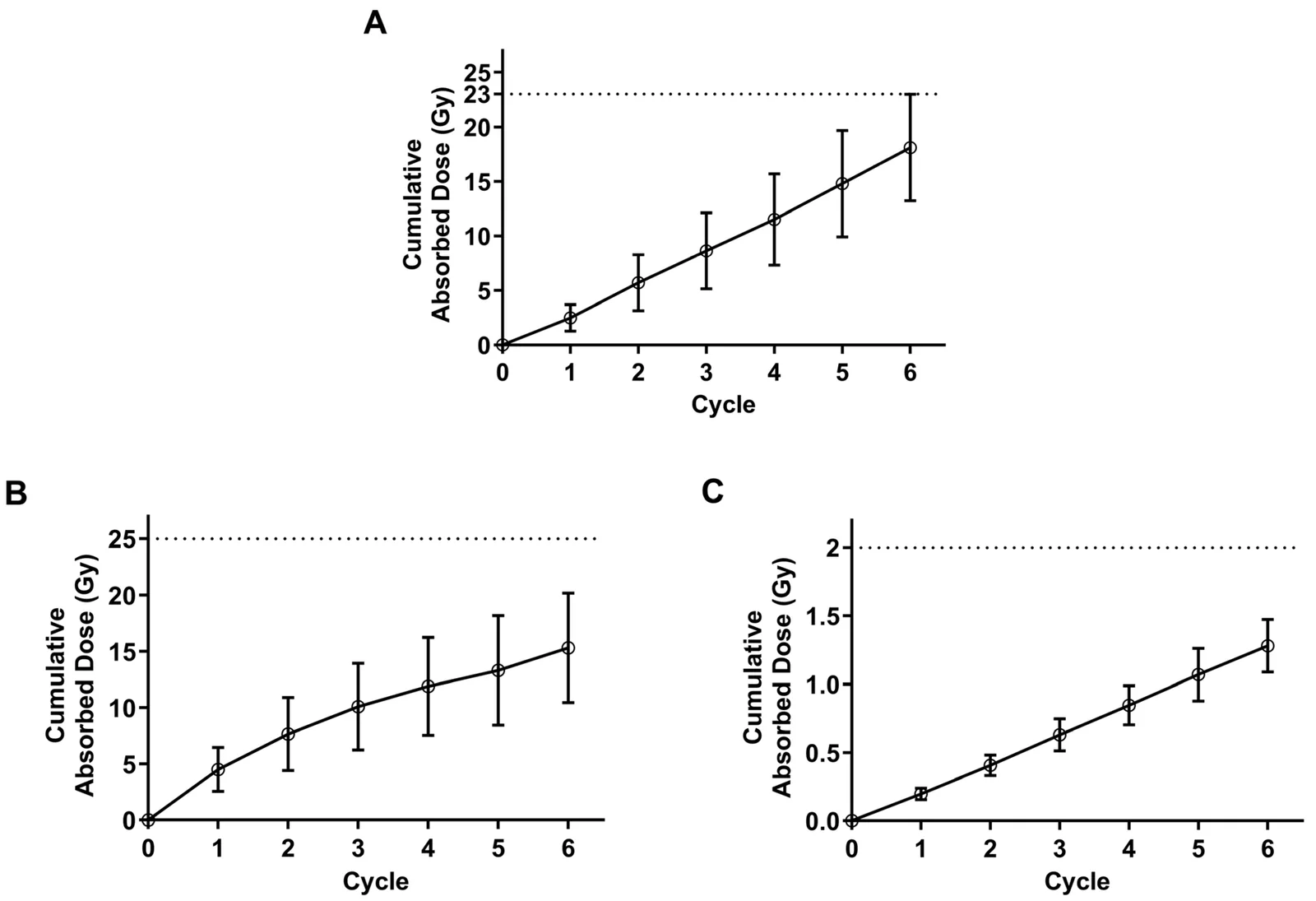
Cumulative absorbed doses (Gy) to the (**A**) kidneys, (**B**) salivary glands, and (**C**) red marrow over six treatment cycles, calculated by summing the cycle-specific mean absorbed dose coefficients through each cycle and multiplying by 3.70 GBq per cycle. The displayed bars represent the arithmetic sums of the available cycle-specific SDs, also multiplied by 3.70 GBq; they are descriptive and do not represent patient-level cumulative-dose SDs, confidence intervals, or statistically propagated uncertainties. No SD contribution could be estimated for cycle 6 (*n* = 1). Dashed lines indicate the reference dose constraints used for comparison.

The sum of the cycle-specific mean absorbed dose coefficients over six cycles of [^177^Lu]Ludotadipep was highest for the kidneys (4.90 Gy/GBq), corresponding to a projected cumulative absorbed dose of 18.1 Gy assuming an administered activity of 3.70 GBq per cycle (Figure 3A). The second highest cumulative absorbed dose coefficient was noted in the salivary glands, with 4.12 Gy/GBq (15.3 Gy/3.70 GBq administration) (Figure 3B). The red marrow cumulative absorbed dose coefficient was 0.346 Gy/GBq (1.28 Gy/3.7 GBq administration) (Figure 3C). No case demonstrated cumulative dose higher than the commonly applied limits of 23 Gy in the kidneys, 25 Gy in the salivary glands, or 2 Gy in the red marrow (Table 2).

## 4. Discussion

In this study, dosimetric analysis was conducted across 67 treatment cycles in 20 patients from the phase 2 trial evaluating [^177^Lu]Ludotadipep, a novel PSMA-targeted radiopharmaceutical with an albumin-binding motif. Accurate red marrow dosimetry was of key interest, given its low tolerance threshold and the potential susceptibility to albumin-bound agents. In an attempt to more accurately reflect the physiology of the albumin binding agent, we applied an HCT-adjusted RMBLR of 0.28 computed from the models established for monoclonal antibodies, and validated the calculations using quantitative SPECT in conjunction with venous blood activity. The resulting red marrow absorbed a dose of 0.0533 ± 0.0115 Gy/GBq for cycle 1, and the cumulative dose of 1.28 Gy over six cycles was below the commonly used 2 Gy safety threshold. The salivary glands and kidneys received the highest absorbed doses in the first cycle, and the salivary gland exposure significantly declined over time (*p* < 0.001). Increasing trends in absorbed doses were observed for the kidneys, liver, and red marrow over time. Overall, the cumulative organ doses remained within accepted safety margins and support the feasibility of repeated administration of [^177^Lu]Ludotadipep.

The absorbed dose coefficients observed in the first cycle of this phase 2 trial were largely consistent with those from the phase 1 study of [^177^Lu]Ludotadipep, corroborating the reproducibility of the dosimetry profile [11]. The mean absorbed doses from the phase 1 single-administration study, and the corresponding values from the first cycle of this phase 2 study were comparable (1.17 ± 0.81 Gy/GBq, 1.22 ± 0.530 Gy/GBq for the salivary glands; 0.77 ± 0.28 Gy/GBq, 0.674 ± 0.330 Gy/GBq for the kidneys; and 0.07 ± 0.02 Gy/GBq, 0.0533 ± 0.0115 Gy/GBq for the red marrow). The alignment between the phase 1 and 2 data corroborates the reproducibility of the dosimetry profile of [^177^Lu]Ludotadipep and supports the selected treatment activity and cycle.

Compared with [^177^Lu]Lu-PSMA-617, which reported mean renal absorbed doses of 0.43 ± 0.16 Gy/GBq at cycle 1 and 15 ± 6 Gy cumulatively over six cycles at 7.4 GBq per cycle, [^177^Lu]Ludotadipep demonstrated a higher kidney absorbed dose coefficient (0.674 ± 0.330 Gy/GBq at cycle 1, and 18.1 ± 4.88 Gy cumulatively over 3.7 GBq × 6 cycles) [26]. Despite the higher absorbed dose coefficient per GBq, the reduced administered activity per cycle with [^177^Lu]Ludotadipep resulted in an overall level of renal exposure that remained within safety limits.

Red marrow is one of the most critical organs in radiopharmaceutical therapy and its radiation tolerance dose is set low, commonly around 2 Gy. If the specific bone uptake is neglected, the absorbed dose to red marrow comprises the self-dose from the marrow regions (red marrow → red marrow) and the cross-dose from surrounding tissues (other tissues → red marrow). Two main approaches used for estimating the self-dose are image-based and blood-based methods [15,21]. Image-based dosimetry typically quantifies radioactivity in trabecular bone regions in the lumbar spine and extrapolates to the total red marrow. However, patients with mAPMR often have multiple metastatic lesions in the bones which limit the applicability of this image-based approach. The blood-based dosimetry method is more commonly used for red marrow dose estimation in TRT, applying an RMBLR value to the measured blood radioactivity. Small-molecule radiopharmaceuticals like [^177^Lu]Lu-PSMA-617 and [^177^Lu]Lu-DOTATATE typically assume an RMBLR of 1.0 which is based on a study comparing blood activity concentration and marrow aspirate activity concentration [23]. However, monoclonal antibodies and other large molecules require a lower RMBLR, e.g., 0.36 with HCT of 0.45, or individually HCT-adjusted values, which would be more consistent with the American Association of Physicists in Medicine’s recommended 0.2–0.4 range [21]. Although [^177^Lu]Ludotadipep is chemically a small molecule, its albumin-binding motif leads to the predominantly albumin-bound form in circulation in vivo. Albumin has roughly half the molecular weight of a monoclonal antibody, and their pharmacokinetics can be assumed to be similar to each other [13,27]. In this study, we applied an RMBLR of 0.28 for [^177^Lu]Ludotadipep based on a HCT-adjusted model for monoclonal antibodies, which was actually developed by adopting the albumin volume data of 0.19 in the red marrow for the RMBLR calculation [12,13,21]. The calculated study-specific RMBLR value of 0.28 was validated in five patients who did not have perceptible bone metastasis in the lumbar vertebrae and posterior iliac regions using two methods: (1) estimation of total marrow activity using blood samples in comparison to the quantitative SPECT-based measurements in lumbar trabecular bone and (2) direct comparison of activity concentration measurements from the superior vena cava versus posterior iliac bone on quantitative SPECT images. Both methods yielded RMBLR estimates consistent with the HCT-adjusted RMBLR value of 0.28.

The bone marrow aspirates largely reflect the fluid components of the marrow composed primarily of blood and extracellular fluid, and do not accurately represent the cellular, stromal or trabecular bony compartments. The assumption of an RMBLR of 1.0 for small peptide radiopharmaceuticals is primarily supported by a study comparing blood and bone marrow aspirate activity concentrations [14]. However, the authors acknowledged that most of the marrow aspirates consisted of blood and marrow-resident cells were low in content. Such result supports an ECF-to-blood relationship, but cannot justify the whole-marrow tissue equivalence to blood. Physiologically, only freely diffusible small molecules like NaI with total body water distribution can truly equilibrate across all marrow compartments [21,28]. In contrast, hydrophilic, charged small-molecule peptide ligands are unlikely to fully diffuse into cells in the marrow space despite their small size, and the RMBLR should reflect the limited ECF distribution rather than assuming uniformity with blood measurements. Given that the albumin-accessible space in red marrow is approximately 84% of the inulin-accessible space, an RMECFF of 0.23, rather than 0.19, may be more appropriate for small peptide ligands [13].

Previous studies for albumin-binding TRT applied various RMBLR values from 0.32 to 1.0, which followed recommendations for either monoclonal antibody or small molecules [5,6,11,29]. Our study also adopted the HCT-adjusted formula for monoclonal antibody, but to our knowledge, this is the first study to validate the RMBLR by using both the blood sample and quantitative SPECT data for an albumin-binding radiopharmaceutical. The use of an RMBLR of 1.0 for albumin-binding TRT would lead to overestimation of marrow absorbed doses and potentially limit the therapeutic dose. Applying an RMBLR biologically tailored for albumin-binding agents would lead to a more accurate quantification of red marrow dosimetry, which would be the foundation for safe dose escalation.

In this study, the absorbed dose coefficient of the salivary glands showed a significant and progressive decline over the treatment cycles. This trend may reflect cumulative radiation-induced damage, which would have led to reduced glandular function and thus lower tracer uptake. Similar patterns have been reported in PSMA-targeted radioligand therapies and are clinically important, given that xerostomia is a common and quality-of-life-impacting adverse effect [26]. These findings highlight the need for better salivary gland-sparing strategies, such as cooling to induce vasoconstriction or the development of protective agents.

This study has several limitations that warrant consideration. First, while 67 cycles were included in the analysis, only a few patients received the fifth and sixth cycles at the time of this assessment. Second, organ dosimetry was primarily based on planar imaging, which is bound to have greater degree of uncertainty due to the limited three-dimensional resolution compared to quantitative SPECT/CT-based methods. However, it should be noted that our phase 2 dosimetry results are consistent with the phase 1 dosimetry results derived from SPECT/CT data. Third, tumor dosimetry is not reported in tsed analysis, preventing a within-cohort assessment of tumor-to-organ absorbed dose ratios or tumor dose–response relationships. A dedicated subsequent report is planned to address tumor dosimetry and treatment efficacy. Fourth, metastatic bone uptakes adjacent to red marrow were not included in the calculation of red marrow absorbed dose, while many of our patients are bound to have bone metastases. Fifth, treatment attrition and the small number of patients evaluated in later cycles may have introduced selection bias, limiting the interpretation of observed changes in organ absorbed dose coefficients across cycles. Finally, while the marrow dosimetry incorporated a biologically relevant RMBLR, we applied the mean RMBLR value of the group, rather than individualized values because validation was done only in a subset of subjects. Physiologically, the RMBLR would vary over time and subjects, but we applied a fixed value to simplify the calculations [30]. Despite these limitations, the findings could provide insight into the dosimetric profile of [^177^Lu]Ludotadipep and support its continued evaluation in larger clinical trials.

## 5. Conclusions

In this phase 2 dosimetry study, [^177^Lu]Ludotadipep showed a safety profile with organ-absorbed doses remaining below the established toxicity thresholds over multiple treatment cycles. Two complementary validation approaches supported the use of a study-specific RMBLR of 0.28 for red marrow dose estimation. Findings from this study could be utilized for future development and clinical use of albumin-binding TRT.

## Figures and Tables

**Table 1:** Organ-specific absorbed dose coefficients (Gy/GBq) per treatment cycle and cumulative values over 6 cycles. For cycle-specific rows, values are mean ± standard deviation (SD), and *n* denotes the number of subjects with evaluable dosimetry in that cycle. In the cumulative row, the first value is the sum of the six cycle-specific means, and the value following ± is the arithmetic sum of the available cycle-specific SDs. This SD sum is descriptive and does not represent the SD of patient-level cumulative dose coefficients.

	Kidney	Liver	Lung	Red Marrow	Salivary Gland	Spleen
Cycle 1 (*n* = 20)	0.674 ± 0.330	0.192 ± 0.0926	0.171 ± 0.0642	0.0533 ± 0.0115	1.22 ± 0.530	0.130 ± 0.0490
Cycle 2 (*n* = 19)	0.867 ± 0.363	0.226 ± 0.0913	0.182 ± 0.0709	0.0567 ± 0.00928	0.847 ± 0.347	0.153 ± 0.0814
Cycle 3 (*n* = 14)	0.792 ± 0.252	0.236 ± 0.0670	0.192 ± 0.0728	0.0602 ± 0.0108	0.652 ± 0.162	0.175 ± 0.131
Cycle 4 (*n* = 8)	0.776 ± 0.191	0.197 ± 0.0411	0.180 ± 0.0360	0.0585 ± 0.00721	0.486 ± 0.136	0.162 ± 0.0700
Cycle 5 (*n* = 5)	0.894 ± 0.184	0.205 ± 0.0672	0.178 ± 0.0255	0.0595 ± 0.0134	0.402 ± 0.142	0.155 ± 0.0953
Cycle 6 (*n* = 1)	0.901	0.195	0.168	0.0582	0.520	0.244
Cumulative absorbed dose coefficient (Gy/GBq)	4.90 ± 1.32	1.25 ± 0.359	1.07 ± 0.269	0.346 ± 0.0522	4.12 ± 1.32	1.02 ± 0.427

**Table 2:** Individual cumulative absorbed doses in kidneys, salivary glands, and red marrow.

Subject	Evaluable Administrations	Cumulative Absorbed Dose (Gy)		
		Kidneys	Salivary Glands	Red Marrow
A01001	2	8.11	6.38	0.37
A01002	5	12.22	11.67	1.37 *
A01003	3	7.73	11.98	0.71
A01004	2	2.35	3.63	0.42
A01005	2	4.34	5.61	0.37
A01006	5	16.48	8.70	0.94
A01007	3	11.39	8.06	0.68
A01008	2	4.34	6.86	0.29
A01009	1	1.98	5.70	0.23
A01010	5	18.95 *	17.92 *	1.04
A01011	2	11.77	7.86	0.42
A01012	6	17.01	14.42	1.33
A01013	4	11.15	13.58	0.71
A01014	3	5.15	5.35	0.70
A01015	3	8.05	12.85	0.60
A01016	5	18.23	16.12	0.89
A01017	4	12.91	16.17	0.83
A01018	3	5.27	14.18	0.59
A01019	3	10.99	11.46	0.66
A01020	4	6.31	8.64	0.95

*, maximum value of cumulative absorbed dose in each organ.

## Data Availability

The datasets presented in this article are not readily available because the clinical trial participants did not consent to data access to third party. Requests to access the datasets should be directed to J.H.O. and C.P.

## Abbreviations

A	activity
cpm	counts per minute
ECF	extracellular fluid
HCT	hematocrit
SUV_mean_	mean standardized uptake value
mAPMR	metastatic androgen pathway modulator-resistant
p.i.	post-injection
PSMA	prostate-specific membrane antigen
RMECFF	red marrow ECF fraction
RMBLR	red marrow-to-blood activity concentration ratio
SVC	superior vena cava
TRT	targeted radionuclide therapy
